# Texture-Modified Soy Protein Gels Using Transglutaminase and Agar for Elderly Dysphagia Management

**DOI:** 10.3390/gels11040303

**Published:** 2025-04-20

**Authors:** Puchcharin Paleekui, Benjamard Rattanamato, Nattapong Kanha, Kanyasiri Rakariyatham, Wannaporn Klangpetch, Sukhuntha Osiriphun, Thunnop Laokuldilok

**Affiliations:** 1 Faculty of Agro-Industry, Chiang Mai University, Chiang Mai 50100, Thailand; puchcharin_pa@cmu.ac.th (P.P.);; 2 Office of Research Administration, Chiang Mai University, Chiang Mai 50200, Thailand; 3 Cluster of High Value Products from Thai Rice and Plants for Health, Chiang Mai University, Chiang Mai 50200, Thailand

**Keywords:** universal design food, texture modification, soy protein isolate, agar, transglutaminase

## Abstract

Dysphagia, a condition characterized by difficulty swallowing, is commonly found in the elderly, increasing the risk of choking and aspiration. This study aimed to develop a texturally modified soy protein gel that meets the Universal Design Food (UDF) standard, specifically for elderly individuals with dysphagia. To create soft-textured foods, the gel’s texture was modified using transglutaminase at varying concentrations (0.5%, 1.0%, and 2.0%, *w*/*v*) in combination with agar. The soy protein gel prepared with 0.5% transglutaminase exhibited the lowest hardness value (2.2 × 10^3^ N/m^2^) and was classified as Level 4 under the UDF standard, indicating that it requires no chewing and is easy to swallow, making it suitable for individuals with severe dysphagia. In contrast, the gel formulated with 2.0% transglutaminase and 0.5% agar had the highest hardness value (3.29 × 10^4^ N/m^2^) and was classified as Level 2, meaning it can be easily mashed with the gums, making it appropriate for individuals with moderate dysphagia. Structural analyses revealed that modifying with transglutaminase altered the protein’s secondary structure by reducing the content of α-helixes and β-sheets while increasing β-turns, potentially enhancing gel network flexibility. These findings suggest that the combined use of transglutaminase and agar effectively modifies soy protein gel texture to meet the dietary needs of elderly individuals with dysphagia. This approach shows promise for the food industry by providing safer and more diverse food options for aging populations facing dysphagia.

## 1. Introduction

Age-related health issues are becoming a global concern as the elderly population continues to grow. Physiological aging, illnesses, and medications cumulatively affect various aspects of oral physiology, including tooth loss, reduced jaw muscle strength, changes in salivary composition, decreased salivary flow, and swallowing disorders such as delayed swallowing response. These factors can contribute to dysphagia, even in otherwise healthy older individuals [1,2].

Dysphagia is characterized by difficulty swallowing due to decreased function or impaired coordination of the muscles of the tongue, mouth, pharynx, and esophagus [3]. This condition can lead to severe health complications, including malnutrition, aspiration pneumonia, and an increased risk of choking due to food or liquid entering the airway—potentially fatal if not addressed promptly [4]. To mitigate these risks, elderly individuals with dysphagia are often prescribed texture-modified foods to ensure safe and convenient consumption. Elderly individuals are typically advised to consume softened, smooth-textured foods that facilitate swallowing by reducing chewiness, incorporating food hydrocolloids, increasing hydration, or decreasing particle size [5]. Texture-modified foods and beverages with enhanced viscosity improve swallowing efficiency, reduce choking risks, and promote safe and enjoyable eating [6,7].

Texture-modified foods are classified based on specific nomenclature and categorization standards. The Universal Design Foods (UDFs) framework, established by the Japan Care Food Conference, categorizes soft foods into four levels according to hardness (N/m^2^):

Levels 1: <5 × 10^5^ N/m^2^ (easy to chew)
Levels 2: <5 × 10^4^ N/m^2^ (can be broken up using the gums)
Levels 3: <2 × 10^4^ N/m^2^ (can be broken up using the tongue)
Levels 4: <5 × 10^3^ N/m^2^ (does not require chewing) [5]

Similarly, the International Dysphagia Diet Standardization Initiative (IDDSI) classifies foods into eight levels (0–7), with Levels 1–4 referring to thickened liquids and Levels 4–7 encompassing texture-modified foods. Spoon tilt and fork tests are employed to assess texture levels and ensure alignment with IDDSI guidelines. IDDSI is widely used for individuals with dysphagia across all age groups and healthcare settings [8,9].

Proteins play a crucial role in addressing nutritional deficiencies in the elderly [10]. Consuming protein above the recommended daily intake can enhance muscle function, prevent chronic diseases, and improve quality of life in aging populations [11]. Currently, the demand for animal protein is increasing in parallel with global population growth. However, the expansion of the animal-based food industry contributes to increased land use for animal husbandry, freshwater consumption, and energy demand while also generating pollution and greenhouse gases. Consequently, plant-based diets are gaining attention as a more sustainable alternative [12,13].

Soy protein, a widely recognized plant-based protein source, serves as a viable alternative to animal proteins due to its comparable nutritional profile and high digestibility [14,15,16]. Especially, soy protein isolate is processed by removing soy pulp and whey from defatted soybean protein (DSP) and contains phenolic compounds and γ-aminobutyric acid, which can contribute to health benefits [17]. Although soy protein can induce allergies in some consumers and cause several immunological symptoms, including urticaria, rhinitis, pruritus, asthma, anaphylactic shock, and even death [18], thermal food processing can destroy some critical epitopes, leading to a reduction in immunoreactivities [18,19,20]. Pure plant-based protein gels frequently have the issue of being overly soft and brittle in texture [21]. Several studies show gel formed by protein and polysaccharide has non-covalent and covalent interactions, thus enhancing the physical properties, mechanical properties, and stability of gel materials [22,23,24,25].

Polysaccharides derived from seaweed are widely utilized in the food industry due to their diverse functional properties. Agar is used in almost 90% of predominantly used foods and has thickening, stabilizing, and gelling properties [26,27]. It also functions as a valuable source of dietary fiber, contributing to digestive health and facilitating intestinal regulation [28]. Moreover, this hydrocolloid is both tasteless and odorless, making it an effective gelling agent that does not alter the flavor profile of the foods to which it is added. The agar gel is the coil-double helix transition, and the interaction creates a 3D network capable of trapping water molecules [29]. When the solvent temperature is over 85 °C, the agar transitions into a solution state. When the temperature ranges between 32 °C and 43 °C, agar undergoes gelation [30]. Moreover, the structure of agar can form at low concentration (0.04% in a solution) [31]. However, the agar gels include brittle and mushy gels, susceptibility to syneresis, and relatively low nutritional values [32,33,34].

Transglutaminase (EC 2.3.2.13), an enzyme recognized as generally safe (GRAS) by the Food and Drug Administration (FDA), is a promising tool for texture modification in the food industry. Transglutaminase is stable across a broad pH range (5.0–8.0), has an optimal temperature of 50 °C, is independent of Ca^2+^, and does not require special cofactors for activation [34,35,36]. Transglutaminase catalyzes covalent bond formation between glutamine and lysine residues in proteins [37,38], leading to improved texture and enhanced functional properties, such as solubility, water-binding capacity, emulsification, foaming, and gelation. Additionally, transglutaminase can enhance the nutritional value of proteins by incorporating essential amino acids [39,40,41].

Several studies show the effect of polysaccharides and transglutaminase on modifying the structure and sensory attributes of plant protein gels [13,42,43]. Therefore, there is a concept that explores the feasibility of using soy protein isolate, transglutaminase, and agar in edible gel. Transglutaminase can be used to modify the texture of soy protein gel to achieve desirable properties, making this enzyme a worthwhile option for the food industry to produce foods to manage dysphagia in the elderly. This approach aligns with the standard of Universal Design Food. The findings from this research guideline for knowledge enhancement can be applied to other plant proteins as more options regarding taste, allergy, and cost become available, and it is possible to test this food on different age groups or health conditions in the future.

## 2. Results and Discussion

### 2.1. Texture Profile Analysis

This study used texture profile analysis (TPA) principles with a texture analyzer to examine prepared soy protein gel samples, simulating human chewing behavior. A two-bite compression test was employed to characterize the textural properties of foods during the chewing process in the oral cavity. The TPA yielded five parameters (hardness, cohesiveness, springiness, gumminess, and chewiness) to describe the texture of foods. The results of the TPA parameters measured are presented in Table 1. The soy protein gel samples, prepared using all seven formulations, exhibited hardness values ranging from 2.2 × 10^3^ to 3.29 × 10^4^ N/m^2^. According to the Universal Design Food standard, these values classify the texture as levels 2 (easy to crush with the gums) to 4 (no need to chew). Therefore, all soy protein gel samples had hardness values suitable for elderly individuals who experience difficulty swallowing.

Initial experiments with soy protein gels prepared without the addition of transglutaminase revealed that soy protein alone could not form a gel. However, when 0.5% transglutaminase was added, soy protein gels formed with a gel hardness of 2.2 × 10^3^ N/m^2^, indicating that enzyme activity is crucial in developing the protein gel structure. Increasing the concentration of transglutaminase to 1.0% resulted in a gel hardness of 4.3 × 10^3^ N/m^2^, but this value did not differ significantly (*p* ≥ 0.05) from the soy protein gel containing 0.5% enzyme. In contrast, a soy protein gel formula using 2.0% transglutaminase produced a hardness of 7.2 × 10^3^ N/m^2^, showing a statistically significant difference (*p* < 0.05) from the previous two formulas with 0.5% and 1.0% enzyme. This suggests that gel hardness tends to increase with higher concentrations of transglutaminase, corroborating findings by [14], which indicated that increased enzyme concentration significantly enhances the strength of pea protein gel. The increased enzyme concentration enhances cross-linking between protein chains [44] and catalyzes acyl transfer reactions between the γ-carbonyl group on glutamic acid and the ε-amino group on lysine [45], leading to the creation of covalent peptide bonds [46]. Furthermore, increasing the transglutaminase concentration from 0.5% to 2.0% also amplified all textural parameters (*p* < 0.05). However, the soy protein gel made with only transglutaminase reached the highest hardness at level 4 and could not be increased to level 3. Developing products that cater to levels 2 to 4 would provide more options for the elderly and individuals with dysphagia. Thus, gelling agents were introduced to assist in preparing soy protein gels in combination with the transglutaminase.

In this study, a 0.5% concentration of agar was used as the gelling agent across all formulations, while the concentration of transglutaminase varied at four levels: 0% (no enzyme added), 0.5%, 1.0%, and 2.0%. The results (Table 1) showed that soy protein gels with only agar had significantly higher hardness (1.26 × 10^4^ N/m^2^) compared to all formulations without agar (2.2–7.2 × 10^3^ N/m^2^) (*p* < 0.05). This finding aligns with the results of [47], who found that the addition of gellan gum significantly enhanced the hardness and other textural parameters of potato protein gels (*p* < 0.05). The addition of agar improves soy protein gel formation, probably due to agar’s ability to integrate well with water and create a more organized agar-soy protein gel structure compared to soy protein gels that lacked agar and relied solely on enzymes for gelation. This improved structure may directly contribute to the increased hardness of the soy protein gels. When preparing soy protein gels using 0.5% agar and 0.5% transglutaminase, the hardness was not significantly different from the formula without transglutaminase (*p* ≥ 0.05). This may be due to the relatively low enzyme concentration, as its activity was insufficient to affect gel hardness. However, increasing the transglutaminase concentration to 1.0% and 2.0% resulted in significantly greater hardness of the prepared soy protein gels (*p* < 0.05). This increase in enzyme concentration facilitated a cross-linking reaction between glutamine and lysine amino acids, leading to the formation of more covalent bonds. Consequently, this reaction produced an interwoven structure of agar and protein chains. As a result, hydrogen bonds may also develop between the agar and protein chains. Therefore, higher concentrations of transglutaminase contributed to a firmer soy protein gel structure.

Additionally, notable changes in cohesiveness and springiness were observed, as shown in Table 1. The cohesiveness of soy protein gel samples prepared with transglutaminase at concentrations of 1.0% and 2.0% did not differ significantly (*p* ≥ 0.05) but was significantly higher than that of gels prepared with 0.5% enzyme (*p* < 0.05). Similarly, the cohesiveness of samples made with 1.0% and 2.0% transglutaminase was not significantly different from those prepared with agar as a gelling agent across all enzyme concentrations (*p* > 0.05). The results indicated that transglutaminase concentration had a relatively minor effect on cohesiveness, showing no significant impact on soy protein gel samples made with agar as the gelling agent. Lower cohesiveness in food products may be advantageous for elderly individuals with dysphagia, as it allows for easier separation and bolus formation during swallowing, benefiting this demographic. The cohesiveness of all soy protein gel samples in this study ranged from 0.35 to 0.59, aligning with the findings of [48], who prepared banana gel with carrageenan as a dessert for elderly individuals with dysphagia, reporting a binding energy range of 0.48 to 0.51.

Regarding springiness (Table 1), no statistically significant difference (*p* > 0.05) was observed between soy protein gel samples prepared with 1.0% and 2.0% transglutaminase. However, these samples exhibited significantly higher springiness (*p* < 0.05) than those prepared with 0.5% transglutaminase. When soy protein gels were made using agar as a gelling agent, samples without transglutaminase had the lowest springiness (*p* < 0.05). In contrast, springiness was significantly higher (*p* < 0.05) when transglutaminase was included. No significant differences were observed in springiness among soy protein gel samples containing agar and varying transglutaminase concentrations (*p* > 0.05). Ref. [48] reported that elderly individuals tend to dislike gel foods with high springiness as they require more chewing and swallowing.

The gumminess and chewiness results are presented in Table 1. An increase in transglutaminase concentration led to a significant rise in gumminess and chewiness in soy protein gel samples (*p* < 0.05). All samples prepared using agar exhibited higher gumminess and chewiness than those without agar. The highest gumminess (*p* < 0.05) was observed in the soy protein gel sample containing agar and 2.0% transglutaminase. Additionally, increasing transglutaminase concentration significantly raised chewiness in soy protein gel samples made with agar (*p* < 0.05). These results indicate that the addition of a gelling agent and transglutaminase concentration influences both texture parameters. Higher values can impact the number of chewing and swallowing cycles required for elderly individuals, potentially increasing the risk of choking [48].

### 2.2. Water Holding Capacity (WHC) and Color Properties

The water-holding capacity (WHC) and color properties of soy protein gel samples are presented in Table 2. The results indicated that the sample containing 0.5% agar and 2.0% transglutaminase demonstrated the highest WHC at 48.7% (*p* < 0.05). This finding aligns with [49], who reported that adding 0.5% agar increased the WHC of casein micelle gels induced by rennet from 63.30% to 92.44%. The sample with the highest WHC exhibited an interwoven structure of agar and protein chains, which enabled it to retain more water. However, the increase in WHC depended on the concentration of transglutaminase. This observation is consistent with the findings of [39], who demonstrated that transglutaminase significantly enhanced the WHC of soy protein isolate emulsion gels. Increasing the transglutaminase concentration from 0 to 5 U/g led to an approximately 20% increase in WHC. WHC is crucial for maintaining a soft and elastic texture, which is particularly important in foods for elderly individuals with dysphagia.

Regarding color properties, soy protein gel samples containing agar and transglutaminase exhibited significantly lower lightness (L*) values than those without agar (*p* < 0.05) (Table 2). Conversely, darker gel samples exhibited higher redness and yellowness values. This phenomenon may be due to residual pigments, such as carotenoids in soy protein. The dense, opaque structure formed by the gel at higher transglutaminase concentrations resulted in reduced lightness. This internal layer of opaque gel served as a background, enhancing the visibility of residual carotenoid pigments in the soy protein gel, which led to increased redness and yellowness.

Furthermore, soy protein gels containing both transglutaminase and agar exhibited significantly higher color difference (∆E*) values than those without agar or with only transglutaminase (*p* < 0.05). This result indicates that the higher color difference reflects the successful texture modification achieved through the transglutaminase’s activity and agar’s gelling properties.

### 2.3. Fork Pressure Tests on Soy Protein Gel Samples

**Figure 1:**
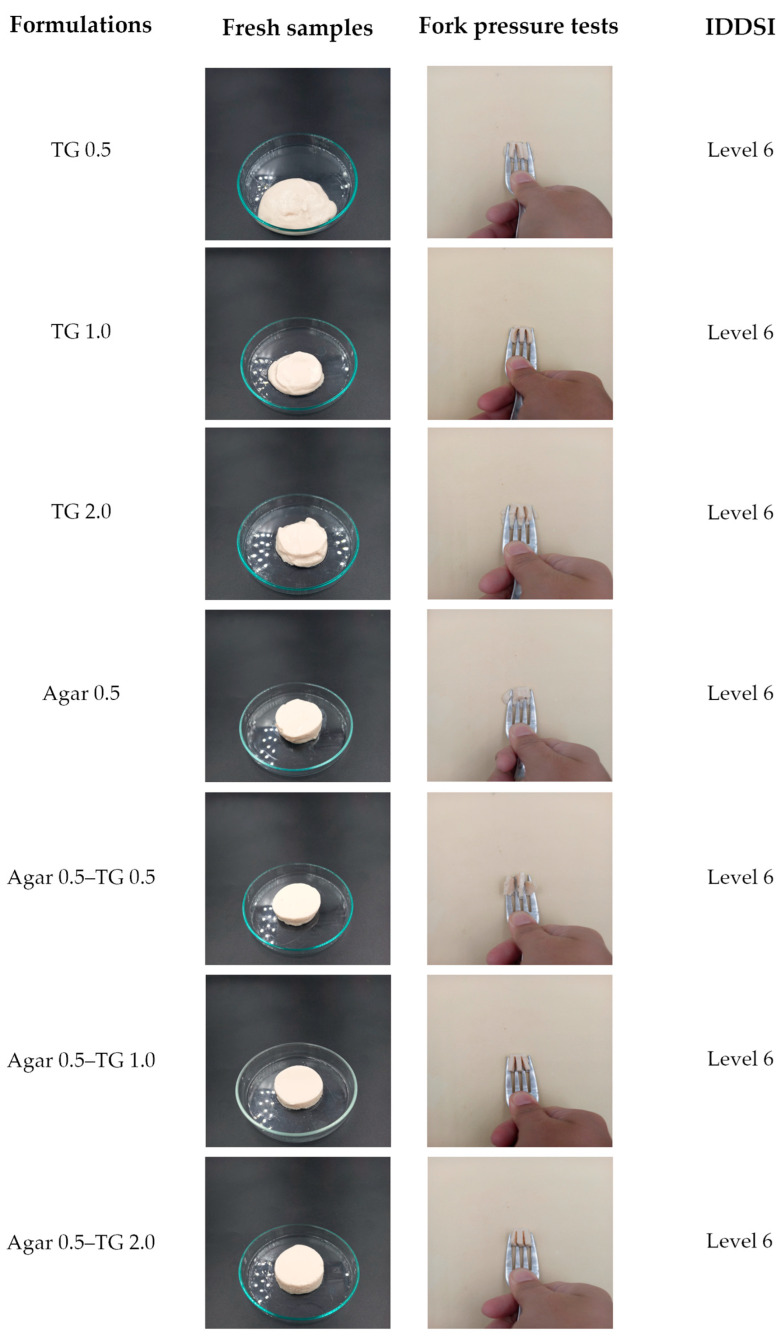
Fork pressure tests on soy protein gel samples followed the International Dysphagia Diet Standardization Initiative (IDDSI).

Fork pressure tests were conducted on soy protein gels to evaluate their suitability as a food option for elderly individuals with dysphagia. Foods deemed safe for swallowing typically range in size from 2 to 4 mm [50,51], which closely aligns with the 4 mm gap of a fork. The test involved using a food cube measuring 1.5 × 1.5 × 1.5 cm, a size that minimizes choking risk [52]. During the test, the thumb was used to press the base of the fork into the gel cube until the nail turned pale, and changes in the samples were observed. As shown in Figure 1, when soy protein gel samples were pressed with a fork, the gel moved in the opposite direction of the applied pressure and flowed up along the fork gap. The size of the gel that flowed up remained consistent with the fork gap, suggesting that the food was appropriately portioned to minimize choking risk. All soy protein gel formulations produced similar results. Based on the assessed characteristics and in accordance with the International Dysphagia Diet Standardization Initiative (IDDSI), the results indicate that all tested soy protein gel formulations are classified as Level 6 (soft and bite-sized).

### 2.4. Fourier Transform Infrared Spectroscopic Analysis of Protein Secondary Structures

**Figure 2:**
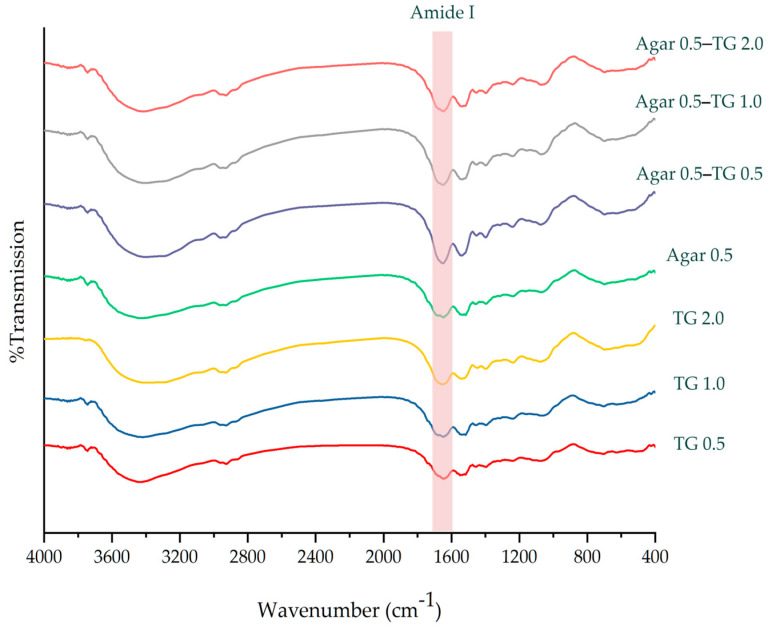
FTIR spectrum of soy protein gels.

Fourier transform infrared spectroscopy (FTIR) is a technique used to detect subtle changes in protein secondary structures [53]. Figure 2 displays the FTIR spectrum of soy protein gel. The Amide I band is commonly used for the quantitative analysis of protein secondary structures, which primarily result from the stretching of C=O double bonds. These structures include α-helix, β-sheet, β-turn, and random coil [54]. This study showed that soy protein gel samples containing both agar and transglutaminase exhibited significantly lower levels of α-helix and β-sheet content compared to samples with either agar or transglutaminase alone (Table 3). The reduction in α-helices, which are highly ordered structures, suggests that the soy protein is unfolding and becoming more disordered [55]. Notably, soy protein gels that contained only agar showed the highest random coil content (*p* < 0.05), as transglutaminase activity was absent. Additionally, a decrease in β-sheet content suggests protein relaxation, consistent with findings reported by [56]. The presence of both transglutaminase and agar simultaneously contributed to the formation of a new protein conformation. The alpha-helix and beta-sheet proteins, unfolded by transglutaminase, may have engaged in intermolecular interactions with agar chains through hydrogen bonds, resulting in an irreversible new protein structure. Consequently, the amounts of both alpha-helix and beta-sheet proteins were reduced. In gel samples containing both agar and transglutaminase, an increase in β-turn content was observed, which contributes to the structural flexibility of the complex. Glutamate and aspartic acid are key components of the β-turn structure. Deamidation caused by transglutaminase likely reorients the protein structure toward a new binding interface [57], aligning with the observed textural effects in soy protein gels containing agar. The use of transglutaminase resulted in gels with increased springiness.

### 2.5. DSC Analysis

**Figure 3:**
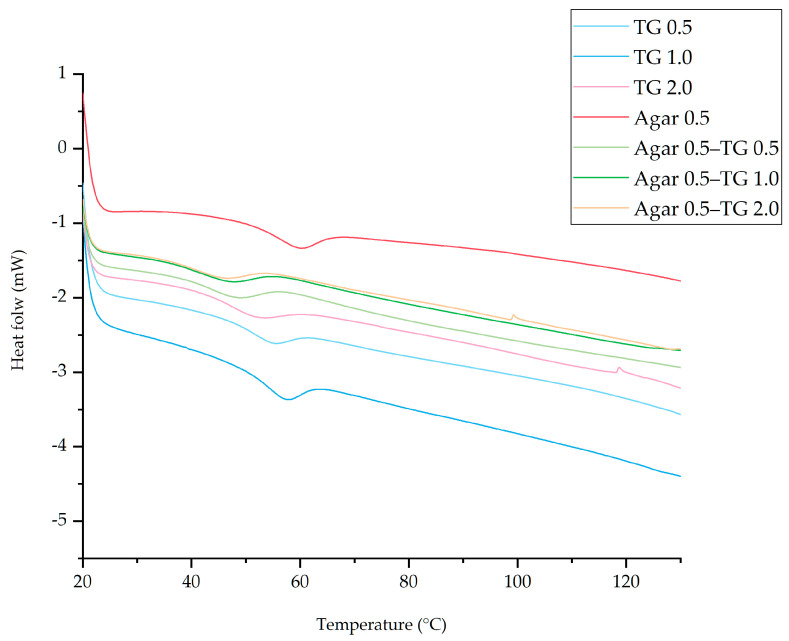
Differential scanning calorimetry (DSC) curves of soy protein gel samples prepared using different concentrations of transglutaminase and agar addition.

The DSC curves (Figure 3) illustrate the thermal properties of soy protein gel samples prepared with varying concentrations of transglutaminase and agar. The results indicate that all samples exhibit a single endothermic transition in their thermograms. Notably, the agar-based soy protein gel that was prepared without transglutaminase treatment showed the highest thermal stability, with onset and peak temperatures recorded at 50.63 °C and 59.48 °C, respectively (Table 4).

For soy protein gels prepared solely with transglutaminase, the highest thermal stability was observed in the sample prepared with 1.0% transglutaminase, with onset (T_0_) and peak temperatures (T_p_) of 49.10 °C and 56.92 °C, respectively. In the agar-based soy protein gel group, the highest glass transition temperature (T_g_) and enthalpy change (ΔH) were observed in the gel prepared without transglutaminase (T_g_ = 54.75 °C, ΔH = 2.65 J/g), followed by gels with 0.5% transglutaminase (T_g_ = 51.25 °C, ΔH = 1.92 J/g), 1.0% transglutaminase (T_g_ = 52.75 °C, ΔH = 2.96 J/g), and 2.0% transglutaminase (T_g_ = 46.75 °C, ΔH = 2.78 J/g). In contrast, among the soy protein gels prepared with agar, the gel prepared with 0.5% transglutaminase exhibited the highest T_g_ and ΔH values (T_g_ = 43.25 °C and ΔH = 2.32 J/g). This was followed by gels with 1.0% transglutaminase (T_g_ = 42.25 °C and ΔH = 2.04 J/g) and 2.0% transglutaminase (T_g_ = 41.25 °C and ΔH = 2.06 J/g). The low T_g_ values indicate reduced thermal stability, suggesting an increased susceptibility to structural denaturation. The thermal stability of the gels is likely influenced by their moisture content, as higher water content generally correlates with lower T_g_ values [58]. The activity of the transglutaminase facilitates the formation of covalent bonds, creating an interwoven structure between protein and agar chains. This denser gel matrix effectively entraps water molecules. Consequently, the agar-based soy protein gel prepared with transglutaminase exhibited lower T_g_ and ΔH values than those prepared with transglutaminase alone. Additionally, the highest T_g_ and ΔH values observed in the agar-based soy protein gel without transglutaminase activity may be attributed to its native soy protein structure, which remained unaltered, facilitating water removal. Although this gel exhibited a high WHC comparable to that of the agar-based soy protein gel containing 2% transglutaminase, it may have experienced greater moisture loss after freeze-drying. Additionally, the T_g_ and ΔH values are affected by the unique unfolding and denaturation properties of proteins, which differ based on the stability, composition, and structure of the protein sources [59].

### 2.6. TGA Analysis

**Figure 4:**
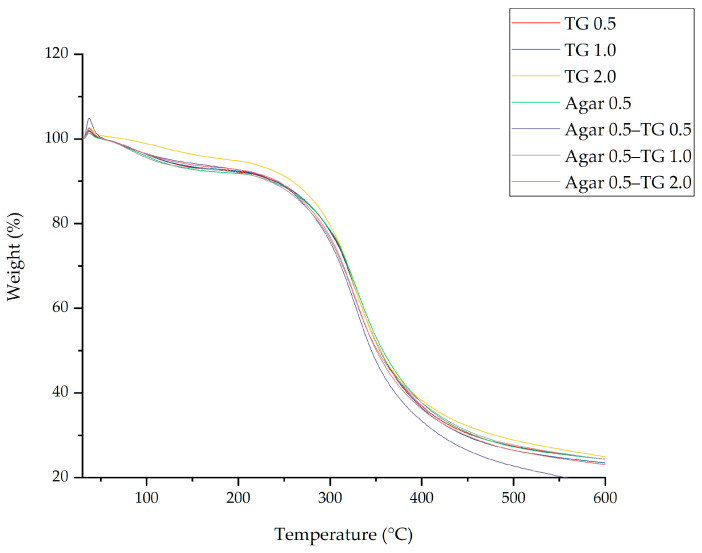
Thermogravimetric analysis curves of soy protein gel samples prepared using different concentrations of transglutaminase and agar addition.

Thermogravimetric analysis (TGA) was performed to assess the thermal stability of soy protein gels by monitoring mass changes during heating from 30 °C to 600 °C. The results revealed two distinct stages of weight loss across all samples (Figure 4).

The initial weight loss, occurring between 50 °C and 150 °C, was primarily due to the evaporation of water. A more significant weight loss occurred in the second stage, beginning around 200 °C, which was linked to the decomposition and disruption of polymer backbones [34]. Among the tested samples, the soy protein gel formulated with 2% transglutaminase exhibited the highest thermal stability, outperforming the gel that contained both 2% transglutaminase and 0.5% agar throughout the entire temperature range. Although the gel with both transglutaminase and agar had a higher hardness, its thermal stability was inferior to that of the gel made solely with transglutaminase. This difference may be attributed to the residual moisture content within the gel. The simultaneous presence of both transglutaminase and agar contributed to the formation of an interwoven protein-agar network, creating a denser gel matrix that retained more water molecules. According to [37], myofibrillar protein gels containing both transglutaminase and κ-carrageenan exhibited greater water retention compared to those treated with transglutaminase alone. It is likely that the water in these structures of gels prepared with transglutaminase and agar is more resistant to complete sublimation. Consequently, the higher moisture content enhances protein chain mobility, rendering the gel more susceptible to thermal denaturation as the temperature rises.

### 2.7. Morphology of Soy Protein Gel Surface and Cross-Section

**Figure 5:**
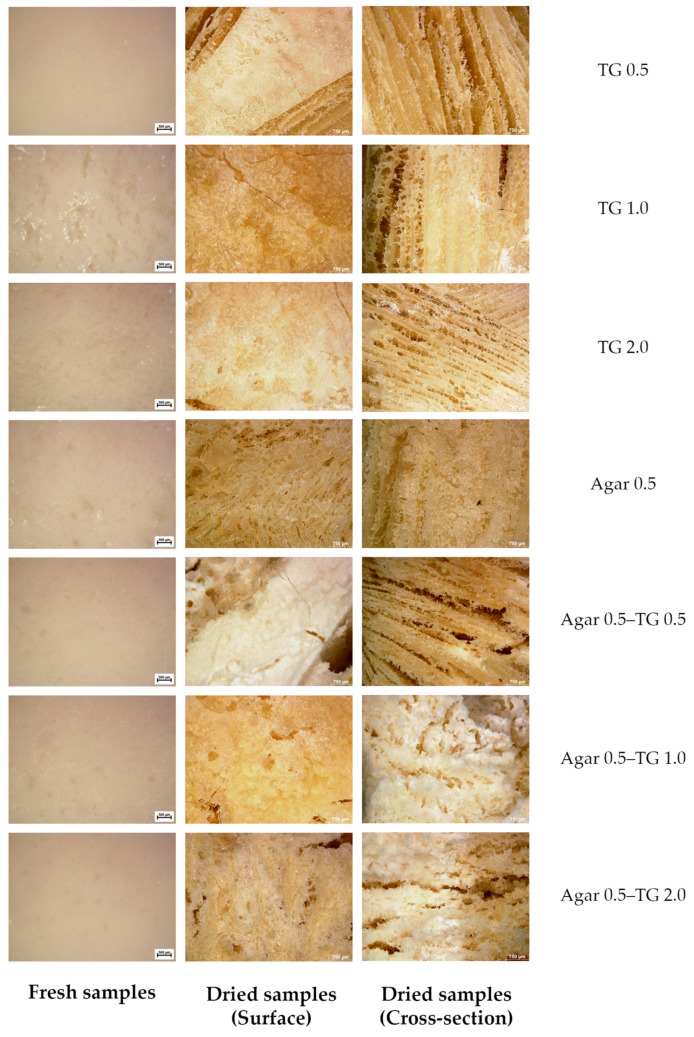
Photographic images of soy protein gel samples prepared using different concentrations of transglutaminase and agar addition.

Figure 5 illustrates the surface and microstructural characteristics of fresh and freeze-dried soy protein gel samples, both on the surface and in cross-section. The fresh gel containing 0.5% transglutaminase exhibited a relatively smooth and homogeneous surface. However, increasing the transglutaminase concentration to 1.0% and 2.0% resulted in a more irregular and uneven surface, likely due to enhanced enzymatic cross-linking and aggregation of soy protein chains. The fresh gel prepared exclusively with agar displayed a smoother surface compared to the control (without agar), except in samples treated with 0.5% transglutaminase. Furthermore, the combination of agar and transglutaminase produced an even smoother surface, suggesting effective synergy in gel formation. Distinct differences in surface and cross-sectional morphology were observed in freeze-dried soy protein gels. The surface of gels containing only transglutaminase was notably less porous than those incorporating both transglutaminase and agar. Cross-sectional analysis revealed considerable differences in gel layer thickness. Soy protein gels formulated with both agar and transglutaminase exhibited a thicker gel layer with a disordered arrangement and significantly fewer and smaller pores, likely due to ice crystal formation during freeze-drying. These characteristics may be attributed to the high moisture content of the gels, where the enzyme-mediated gel network entraps water molecules and limits water sublimation. The findings highlight the role of transglutaminase in cross-linking protein chains and integrating soy protein with agar, thereby reinforcing the gel structure.

## 3. Conclusions

In this study, texture-modified soy protein gels were successfully developed using agar and transglutaminase. Increasing the transglutaminase concentration in the gel formulation led to a proportional increase in gel hardness. Additionally, the incorporation of agar significantly enhanced gel hardness and modified the protein’s secondary structure by reducing α-helix and β-sheet content while increasing β-turn content. Soy protein gels formulated with agar and 2.0% transglutaminase exhibited high gel hardness and water-holding capacity and were classified as Level 2 (easily crushed with gums) according to the Universal Design Food standards. In contrast, gels containing 0.5% or 1.0% transglutaminase displayed lower hardness values, placing them in level 4 (requiring no chewing and suitable for direct swallowing) under the same classification system. Furthermore, all soy protein gels produced in this study were categorized as level 6 (soft and bite-sized) according to the International Dysphagia Diet Standardization Initiative (IDDSI). However, differential scanning calorimetry (DSC) and thermogravimetric analysis (TGA) revealed that while transglutaminase modification improved gel texture and water retention, it also reduced thermal stability. Digital microscopy imaging further corroborated these findings, confirming the structural characteristics associated with the gels’ thermal behavior. The study suggests that combining transglutaminase with agar is an effective strategy for modifying the texture of protein gels. This approach meets the textural requirements of UDF levels 2–4, enhancing the safety of the gels and increasing the variety of food options available for elderly individuals with dysphagia. In addition, these findings can be used as data for the food industry to produce commercial food products that respond to the specific requirements of dysphagic patients and might also be good, healthy foods for other age groups.

## 4. Materials and Methods

### 4.1. Materials

Soy protein isolate (SPI, 86.58% protein) was purchased from Matell Intertrade Co., Ltd. (Khon Kaen, Thailand), and food-grade agar was obtained from Seng Huad Company Limited (Bangkok, Thailand). Transglutaminase (TGase; ACTIVA TG-B) was procured from Ajinomoto Co. (Ajinomoto, Tokyo, Japan), with an enzyme activity of 50 U/g.

### 4.2. Protein Gel Preparation

The soy protein solution was prepared by dissolving powdered SPI in water at a concentration of 15% (*w*/*v*). This mixture was then heated to 90 °C and maintained at this temperature for 10 min. Agar (0% or 0.5%) was then added to the solution and continuously stirred for 10 min. TGase treatment was performed by adding the enzyme to the solution within the temperature range of 40–50 °C, followed by mixing for an additional 5 min. The solutions were transferred to sealed cups and incubated in a water bath (H20 S, Lauda, Germany) at 50 °C for 90 min. All samples were cooled in an ice bath and stored in sealed containers at 4 °C overnight to ensure complete gel setting before analysis to allow the gel formation.

### 4.3. The Universal Design Food (UDF) Analysis by Textural Profile Analysis

The texture characteristics of the gels were evaluated using the Universal Design Food (UDF) technique. Samples at room temperature were cut and placed in a container measuring 40 mm in diameter and 15 mm in height. A texture analyzer (CT3, AMETEK Brookfield Texture, Middleborough, MA, USA), equipped with a 5000 N load cell and a 20 mm diameter cylindrical probe, was used for analysis. The probe was set to compress the samples twice at a speed of 10 mm/s to 30% of their original height. Hardness values were calculated from the maximum force recorded on the force curve and expressed in units of N/m^2^. Additionally, cohesiveness, springiness, gumminess, and chewiness were also recorded.

### 4.4. Water Holding Capacity (WHC)

The water holding capacity (WHC) of the gels was determined according to the method described by [60]. Briefly, 1–2 g of gel sample was placed on Whatman filter paper No. 4 inside a Falcon tube and centrifuged (FC5714 Frontier, OHAUS, Parsippany, NJ, USA) at 5000× *g* for 10 min. After centrifugation, the samples were removed from the filter paper, and the mass of the wet filter paper was recorded. WHC values were calculated using the following Equation (1):

WHC (%) = 100 − [(mf − mi)/ms] 100(1)

where mf is the mass of the wet filter paper (g), mi is the initial mass of the dry filter paper (g), and ms is the mass of the gel sample (1–2 g). All systems were analyzed in triplicate.

### 4.5. Color Analysis

Color properties were determined using a Chroma meter (Konica Minolta: Model CR-400, Japan). The parameters L* (lightness, white to black), a* (redness to greenness), and b* (yellowness to blueness) were used to characterize the sample colors [61]. The total color change (ΔE*) was calculated using the following Equation (2):

(2) $$∆E*=\sqrt{{\left(L^{*}-L_{0}^{*}\right)}^{2}+{\left(a^{*}-a_{0}^{*}\right)}^{2}+{\left(b^{*}-b_{0}^{*}\right)}^{2}}$$

where $L_{0}^{*}$, $a_{0}^{*}$, and $b_{0}^{*}$ represent the color values of the white standard board (L* = 97.6, a* = −0.05, and b* = −1.72, respectively).

### 4.6. Fork Pressure Tests

Fork pressure tests were performed according to the International Dysphagia Diet Standardization Initiative (IDDSI) on sample cubes (1.5 cm × 1.5 cm × 1.5 cm). The shape change of the pressed samples and the color of the thumb were observed under standard lighting conditions [62].

### 4.7. Fourier-Transform Infrared Spectroscopy (FTIR)

Freeze-dried samples were ground and blended with potassium bromide (KBr) powder, according to [14]. The mixture was pressed into tablets and analyzed using a spectrometer (FT/IR-6X/8X High-end model, JASCO, Japan). Spectra were collected at room temperature in the infrared spectral range of 600–4000 cm^−1^ with a resolution of 4 cm^−1^.

### 4.8. Amide I Peak Deconvolution

The analysis of FTIR spectra was carried out using OriginPro 2018 (OriginLab Corporation, Northampton, MA, USA). Peak positions were determined after performing baseline correction on the amide I peak within the range of 1595–1705 cm^−1^. Hidden peaks were identified using a second derivative method. A Gaussian function was applied for the deconvolution and fitting of the peaks. The deconvolution spectrum was shown in Figure S1. Protein secondary structure was evaluated based on the resulting secondary structure components. The percentage of each secondary structure component was calculated by dividing the area of an individual amide I peak by the total area of all amide peak components.

### 4.9. Thermogravimetric Analysis (TGA)

TGA was conducted according to [63], using a TGA analyzer (TGA/DSC3+, Mettler Toledo, Greifensee, Switzerland). Approximately 8–10 mg of the sample was accurately weighed and placed in the analyzer at room temperature. The nitrogen flow rate was set to 40 mL/min, and the temperature increased from 30 to 600 °C at a rate of 20 °C/min.

### 4.10. Differential Scanning Calorimetry (DSC)

The thermodynamic properties of the complex were determined using a differential scanning calorimeter (DSC823e, Mettler Toledo, Greifensee, Switzerland) according to the previously described method [56]. Briefly, 4.0–5.0 mg of the sample was weighed into an aluminum plate and sealed by pressing. An empty plate was used as the control group. The samples were equilibrated and tested at room temperature with a nitrogen flow rate of 40 mL/min, a temperature range of 20 °C to 130 °C, and a heating rate of 10 °C/min.

### 4.11. Digital Microscopes for 2D and 3D Imaging and Analysis

Samples were analyzed using a digital microscope (DVM6, Leica, Wetzlar, Germany) to examine microstructures and surface textures. Microstructural images were captured at 50× magnification using both fresh and freeze-dried samples.

### 4.12. Statistical Analysis

All data are presented as mean ± SD and analyzed using one-way analysis of variance (ANOVA). Significant differences between means were determined using Duncan’s multiple range test at a 95% confidence level (*p* < 0.05), conducted with SPSS version 17 (SPSS Inc., Chicago, IL, USA).

## Figures and Tables

**Table 1:** Texture profile analysis of soy protein gels prepared using various concentrations of transglutaminase (TG).

Formulations	UDF Level	Hardness (N/m^2^)	Cohesiveness	Springiness (mm)	Gumminess (g)	Chewiness (mJ)
TG 0.5	4	2.2 × 10^3^ ± 2.7 × 10^2 e^	0.35 ± 0.04 ^b^	2.21 ± 0.17 ^c^	26.0 ± 4.0 ^e^	0.45 ± 0.05 ^f^
TG 1.0	4	4.3 × 10^3^ ± 1.8 × 10^2 e^	0.51 ± 0.08 ^a^	3.98 ± 0.30 ^a^	73.7 ± 7.5 ^d^	3.0 ± 0.4 ^e^
TG 2.0	4	7.2 × 10^3^ ± 8.3 × 10^2 d^	0.51 ± 0.05 ^a^	3.87 ± 0.29 ^a^	116 ± 10 ^c^	5.1 ± 0.4 ^d^
Agar 0.5	3	1.26 × 10^4^ ± 0.86 × 10^3 c^	0.59 ± 0.04 ^a^	2.92 ± 0.49 ^b^	238 ± 29 ^b^	5.3 ± 0.4 ^d^
Agar 0.5–TG 0.5	3	1.47 × 10^4^ ± 0.45 × 10^3 c^	0.52 ± 0.04 ^a^	3.53 ± 0.20 ^a^	253 ± 12 ^b^	7.6 ± 0.9 ^c^
Agar 0.5–TG 1.0	3	1.93 × 10^4^ ± 2.1 × 10^3 a^	0.52 ± 0.07 ^a^	3.81 ± 0.36 ^a^	259 ± 19 ^b^	10.5 ± 1.3 ^b^
Agar 0.5–TG 2.0	2	3.29 × 10^4^ ± 3.4 × 10^3 a^	0.51 ± 0.06 ^a^	3.79 ± 0.44 ^a^	536 ± 378 ^a^	20.0 ± 1.4 ^a^

Values show the average ± standard deviation of the triplicates. Different superscript letters in each column indicate significant differences at *p* < 0.05.

**Table 2:** Water holding capacity and color properties of soy protein gels prepared using various transglutaminase (TG) concentrations.

Formulations	Water Holding Capacity (%)	Color Parameters			
		L*	a*	b*	∆E*
TG 0.5	39.1 ± 0.9 ^e^	78.32 ± 0.27 ^a^	0.85 ± 0.17 ^d^	12.9 ± 0.3 ^d^	34.8 ± 0.7 ^d^
TG 1.0	40.0 ± 2.6 ^de^	77.67 ± 0.14 ^a^	0.77 ± 0.01 ^d^	12.86 ± 0.05 ^d^	35.33 ± 0.13 ^d^
TG 2.0	42.0 ± 1.2 ^cd^	77.6 ± 0.7 ^a^	1.32 ± 0.06 ^c^	14.6 ± 0.2 ^c^	37.7 ± 0.5 ^c^
Agar 0.5	44.8 ± 1.5 ^b^	75.08 ± 0.09 ^b^	1.90 ± 0.05 ^b^	16.8 ± 0.3 ^b^	43.0 ± 0.2 ^b^
Agar 0.5–TG 0.5	42.3 ± 1.1^bcd^	72.5 ± 0.7 ^e^	2.49 ± 0.16 ^a^	17.2 ± 0.6 ^ab^	46.6 ± 0.4 ^a^
Agar 0.5–TG 1.0	44.3 ± 1.2 ^bc^	73.44 ± 0.30 ^c^	2.58 ± 0.05 ^a^	17.50 ± 0.39 ^a^	46.01 ± 0.38^a^
Agar 0.5–TG 2.0	48.7 ± 0.8 ^a^	73.0 ± 0.6 ^cd^	2.61 ± 0.16 ^a^	17.10 ± 0.39 ^ab^	46.1 ± 0.7 ^a^

Values show the average ± standard deviation of the triplicates. Different superscript letters in each column indicate significant differences at *p* < 0.05.

**Table 3:** Secondary structure (%) of proteins in soy protein gels.

Formulations	β-Sheet		Random Coil	α-Helix	β-Turn	
	1611	1626 ^ns^	1642	1657	1673 ^ns^	1688
TG 0.5	23.7 ± 0.9 ^ab^	25.1 ± 1.0	6.76 ± 0.01 ^e^	5.77 ± 0.01 ^c^	20.6 ± 1.6	18.1 ± 1.7 ^bcd^
TG 1.0	24.3 ± 0.4 ^a^	24.7 ± 0.7	7.41 ± 0.01 ^b^	6.10 ± 0.01 ^b^	20.0 ± 1.3	17.5 ± 1.6 ^cd^
TG 2.0	23.9 ± 1.0 ^ab^	22.8 ± 1.3	7.17 ± 0.01 ^d^	6.11 ± 0.02 ^b^	20.1 ± 1.9	20.5 ± 1.2 ^abc^
Agar 0.5	24.1 ± 0.8 ^a^	25.5 ± 0.3	7.54 ± 0.01 ^a^	6.31 ± 0.01 ^a^	19.7 ± 1.7	16.9 ± 1.7 ^d^
Agar 0.5–TG 0.5	21.0 ± 1.5 ^b^	21.8 ± 3.3	7.19 ± 0.02 ^d^	4.09 ± 0.01 ^f^	22.32 ± 0.08	23.60 ± 0.35 ^a^
Agar 0.5–TG 1.0	22.6 ± 2.0 ^ab^	21.8 ± 3.3	7.27 ± 0.01 ^c^	4.87 ± 0.01 ^e^	20.8 ± 1.3	21.0 ± 0.8 ^ab^
Agar 0.5–TG 2.0	23.2 ± 1.0 ^ab^	24.1 ± 0.8	7.20 ± 0.00 ^d^	5.57 ± 0.01 ^d^	20.7 ± 1.0	19.2 ± 2.4 ^bcd^

Values show the average ± standard deviation of the triplicates. Different superscript letters in each column indicate significant differences at *p* < 0.05; ns = not significantly different (*p* ≥ 0.05).

**Table 4:** The Differential Scanning Calorimetry of soy protein gel.

Formulations	T_0_ (°C)	∆H (J/g)	T_p_ (°C)	T_g_ (°C)
TG 0.5	47.61 ± 0.56 ^b^	1.92 ± 0.11 ^d^	55.19 ± 0.16 ^b^	51.25 ± 0.35 ^c^
TG 1.0	49.10 ± 0.93 ^ab^	2.96 ± 0.05 ^a^	56.92 ± 0.20 ^b^	52.75 ± 0.35 ^b^
TG 2.0	42.37 ± 0.77 ^c^	2.78 ± 0.04 ^ab^	51.48 ± 0.64 ^c^	46.75 ± 0.35 ^d^
Agar 0.5	50.63 ± 0.93 ^a^	2.65 ± 0.016 ^b^	59.48 ± 0.69 ^a^	54.75 ± 0.35 ^a^
Agar 0.5–TG 0.5	38.93 ± 0.14 ^d^	2.32 ± 0.13 ^c^	48.09 ± 0.21 ^d^	43.25 ± 0.35 ^e^
Agar 0.5–TG 1.0	37.01 ± 0.81 ^e^	2.04 ± 0.08 ^d^	46.40 ± 0.54 ^d^	42.25 ± 0.35 ^e^
Agar 0.5–TG 2.0	36.26 ± 0.53 ^e^	2.06 ± 0.11 ^d^	46.98 ± 1.73 ^d^	41.25 ± 0.35 ^f^

Values show the average ± standard deviation of the triplicates. Different superscript letters in each column indicate significant differences at *p* < 0.05.

## Data Availability

The original contributions presented in this study are included in the article. Further inquiries can be directed to the corresponding author.

## Supplementary Materials

The following supporting information can be downloaded at: https://www.mdpi.com/article/10.3390/gels11040303/s1, Figure S1: FTIR and deconvolution spectra in the amide I region of soy protein gels.

## References

[1] Vandenberghe-Descamps M., Labouré H., Septier C., Feron G., Sulmont-Rossé C. (2018). Oral comfort: A new concept to understand elderly people’s expectations in terms of food sensory characteristics. Food Qual. Prefer..

[2] Wang J., Na X., Navicha W.B., Wen C., Ma W., Xu X., Wu C., Du M. (2020). Concentration-dependent improvement of gelling ability of soy proteins by preheating or ultrasound treatment. LWT.

[3] Roldan-Vasco S., Orozco-Duque A., Orozco-Arroyave J.R. (2025). Dysphagia screening with sEMG, accelerometry and speech: Multimodal machine and deep learning approaches. Biomed. Signal Process. Control.

[4] Li M., Wang Z., Han W.-J., Lu S.-Y., Fang Y.-Z. (2015). Effect of feeding management on aspiration pneumonia in elderly patients with dysphagia. Chin. Nurs. Res..

[5] Chao C., Lee J.H., Kim I.W., Choi R.Y., Kim H.W., Park H.J. (2023). Investigation of 3D-printable chickpea-mealworm protein mixtures and their bolus rheology: A soft-textured and safe-swallowing food for the elderly. Food Biosci..

[6] Cichero J.A. (2013). Thickening agents used for dysphagia management: Effect on bioavailability of water, medication and feelings of satiety. Nutr. J..

[7] Hansen T., Beck A.M., Kjaersgaard A., Poulsen I. (2022). Second update of a systematic review and evidence-based recommendations on texture modified foods and thickened liquids for adults (above 17 years) with oropharyngeal dysphagia. Clin. Nutr. ESPEN.

[8] The International Dysphagia Diet Standardisation Initiative IDDSI Framework Testing Methods 2.0. https://www.iddsi.org/images/Publications-Resources/DetailedDefnTestMethods/English/V2TestingMethodsEnglish31july2019.pdf.

[9] Wang X., Chen Y., Dong M., Chen J. (2024). Comparisons of shear and extensional rheological properties of Tremella polysaccharide with commercial thickeners at different IDDSI levels for dysphagia management. Food Hydrocoll..

[10] García J., Méndez D., Álvarez M., Sanmartin B., Vázquez R., Regueiro L., Atanassova M. (2019). Design of novel functional food products enriched with bioactive extracts from holothurians for meeting the nutritional needs of the elderly. LWT.

[11] Baum J.I., Kim I.-Y., Wolfe R.R. (2016). Protein consumption and the elderly: What is the optimal level of intake?. Nutrients.

[12] Ou M., Lou J., Lao L., Guo Y., Pan D., Yang H., Wu Z. (2023). Plant-based meat analogue of soy proteins by the multi-strain solid-state mixing fermentation. Food Chem..

[13] Zhou H., Hu X., Xiang X., McClements D.J. (2023). Modification of textural attributes of potato protein gels using salts, polysaccharides, and transglutaminase: Development of plant-based foods. Food Hydrocoll..

[14] Li T., Zhang J., Hu A., Guo F., Zhou H., Wang Q. (2024). Effect of transglutaminase and laccase on pea protein gel properties compared to that of soybean. Food Hydrocoll..

[15] Tiong A.Y.J., Crawford S., Jones N.C., McKinley G.H., Batchelor W., van ’t Hag L. (2024). Pea and soy protein isolate fractal gels: The role of protein composition, structure and solubility on their gelation behaviour. Food Struct..

[16] Qin P., Wang T., Luo Y. (2022). A review on plant-based proteins from soybean: Health benefits and soy product development. J. Agric. Food Res..

[17] Zhang Y., Chang S.K. (2022). Color and texture of surimi-like gels made of protein isolate extracted from catfish byproducts are improved by washing and adding soy whey. J. Food Sci..

[18] Xia J., Zu Q., Yang A., Wu Z., Li X., Tong P., Yuan J., Wu Y., Fan Q., Chen H. (2019). Allergenicity reduction and rheology property of Lactobacillus-fermented soymilk. J. Sci. Food Agric..

[19] Pi X., Sun Y., Fu G., Wu Z., Cheng J. (2021). Effect of processing on soybean allergens and their allergenicity. Trends Food Sci. Technol..

[20] Zheng H., Yan G., Lee Y., Alcaraz C., Marquez S., de Mejia E.G. (2020). Effect of the extrusion process on allergen reduction and the texture change of soybean protein isolate-corn and soybean flour-corn mixtures. Innov. Food Sci. Emerg. Technol..

[21] Sridharan S., Meinders M.B., Sagis L.M., Bitter J.H., Nikiforidis C.V. (2022). Starch controls brittleness in emulsion-gels stabilized by pea flour. Food Hydrocoll..

[22] Fan Z., Cheng P., Zhang P., Zhang G., Han J. (2022). Rheological insight of polysaccharide/protein based hydrogels in recent food and biomedical fields: A review. Int. J. Biol. Macromol..

[23] Thivya P., Gururaj P., Reddy N.B.P., Rajam R. (2024). Recent advances in protein-polysaccharide based biocomposites and their potential applications in food packaging: A review. Int. J. Biol. Macromol..

[24] Choi M., Choi H.W., Kim H.E., Hahn J., Choi Y.J. (2023). Mimicking animal adipose tissue using a hybrid network-based solid-emulsion gel with soy protein isolate, agar, and alginate. Food Hydrocoll..

[25] Lee S., Jo K., Kim S., Woo M., Choi Y.-S., Jung S. (2025). Exploring the potential of the agar-based emulsion gel as a pork fat substitute in sausage with a focus on the digestive behaviors of lipids and proteins in vitro. Food Hydrocoll..

[26] Ścieszka S., Klewicka E. (2019). Algae in food: A general review. Crit. Rev. Food Sci. Nutr..

[27] Rhein-Knudsen N., Ale M.T., Meyer A.S. (2015). Seaweed hydrocolloid production: An update on enzyme assisted extraction and modification technologies. Mar. Drugs.

[28] Suresh A., Shobna, Salaria M., Morya S., Khalid W., Afzal F.A., Khan A.A., Safdar S., Khalid M.Z., Mukonzo Kasongo E.L. (2024). Dietary fiber: An unmatched food component for sustainable health. Food Agric. Immunol..

[29] Zhang G., Wang Y., Cui P., Qiu Y., Ye S., Zhang A. (2024). pH-induced complex coacervation of Gel and Agar: Phase behavior and structural properties. Process Biochem..

[30] Lee W.-K., Lim Y.-Y., Leow A.T.-C., Namasivayam P., Abdullah J.O., Ho C.-L. (2017). Factors affecting yield and gelling properties of agar. J. Appl. Phycol..

[31] Krasulya O.N., Dunchenko N.I., Yankovskaya V.S., Voloshina E.S., Mettu S. (2022). The effects of ultrasonic treated whey on the structure formation in food systems based on whey in combination with pectin and agar-agar. Ultrason. Sonochem..

[32] Banerjee S., Bhattacharya S. (2011). Compressive textural attributes, opacity and syneresis of gels prepared from gellan, agar and their mixtures. J. Food Eng..

[33] Banerjee S., Ravi R., Bhattacharya S. (2013). Textural characterisation of gellan and agar based fabricated gels with carrot juice. LWT-Food Sci. Technol..

[34] Liu H., Nardin C., Zhang Y. (2024). Novel soft food gels using beta-lactoglobulin via enzymatic crosslinking as agar gel alternatives. Food Hydrocoll..

[35] Amirdivani S., Khorshidian N., Fidelis M., Granato D., Koushki M.R., Mohammadi M., Khoshtinat K., Mortazavian A.M. (2018). Effects of transglutaminase on health properties of food products. Current Opin. Food Sci..

[36] Gaspar A.L.C., de Góes-Favoni S.P. (2015). Action of microbial transglutaminase (MTGase) in the modification of food proteins: A review. Food Chem..

[37] Feng Y., Liang X., Zhang J., Kong B., Shi P., Cao C., Zhang H., Liu Q., Zhang Y. (2024). Effects of transglutaminase coupled with κ-carrageenan on the rheological behaviours, gel properties and microstructures of meat batters. Food Hydrocoll..

[38] Vijayan P., Song Z., Toy J.Y.H., Yu L.L., Huang D. (2024). Effect of transglutaminase on gelation and functional proteins of mung bean protein isolate. Food Chem..

[39] Luo K., Liu S., Miao S., Adhikari B., Wang X., Chen J. (2019). Effects of transglutaminase pre-crosslinking on salt-induced gelation of soy protein isolate emulsion. J. Food Eng..

[40] Yang J., Zhu B., Dou J., Li X., Tian T., Tong X., Wang H., Huang Y., Li Y., Qi B. (2023). Structural characterization of soy protein hydrolysates and their transglutaminase-induced gelation properties. LWT.

[41] Yang X., Zhang Y. (2019). Expression of recombinant transglutaminase gene in Pichia pastoris and its uses in restructured meat products. Food Chem..

[42] Wang K., Wang J., Chen L., Hou J., Lu F., Liu Y. (2024). Effect of sanxan as novel natural gel modifier on the physicochemical and structural properties of microbial transglutaminase-induced mung bean protein isolate gels. Food Chem..

[43] Tay J.U., Oh J.L.-E., Lu Y., Antipina M.N., Zhou W., Huang D. (2024). 3D printing of prawn mimics with faba proteins: The effects of transglutaminase and curdlan gum on texture. Int. J. Biol. Macromol..

[44] Santhi D., Kalaikannan A., Malairaj P., Arun Prabhu S. (2017). Application of microbial transglutaminase in meat foods: A review. Crit. Rev. Food Sci. Nutr..

[45] Herz E., Schäfer S., Terjung N., Gibis M., Weiss J. (2021). Influence of transglutaminase on glucono-δ-lactone-induced soy protein gels. ACS Food Sci. Technol..

[46] Tanger C., Müller M., Andlinger D., Kulozik U. (2022). Influence of pH and ionic strength on the thermal gelation behaviour of pea protein. Food Hydrocoll..

[47] Ryu J., McClements D.J. (2024). Impact of heat-set and cold-set gelling polysaccharides on potato protein gelation: Gellan gum, agar, and methylcellulose. Food Hydrocoll..

[48] Suebsaen K., Suksatit B., Kanha N., Laokuldilok T. (2019). Instrumental characterization of banana dessert gels for the elderly with dysphagia. Food Biosci..

[49] Zhang Y., Wang X., Zhu H., Chen B., Wang C., Pang X., Wang Y., Xie N., Su S., Zhang S. (2024). The effect of agar on rheological properties and thermal stability of rennet-induced casein micelle gel. Colloids Surf. A Physicochem. Eng. Asp..

[50] Peyron M.-A., Mishellany A., Woda A. (2004). Particle size distribution of food boluses after mastication of six natural foods. J. Dent. Res..

[51] Woda A., Nicolas E., Mishellany-Dutour A., Hennequin M., Mazille M.-N., Veyrune J.-L., Peyron M.-A. (2010). The masticatory normative indicator. J. Dent. Res..

[52] Murdan S. (2011). Transverse fingernail curvature in adults: A quantitative evaluation and the influence of gender, age, and hand size and dominance. Int. J. Cosmet. Sci..

[53] Farhat I.A., Orset S., Moreau P., Blanshard J.M. (1998). FTIR study of hydration phenomena in protein–sugar systems. J. Colloid Interface Sci..

[54] Wang J., Xu Z., Jiang L., Zhang Y., Sui X. (2023). Further evaluation on structural and antioxidant capacities of soy protein isolate under multiple freeze–thaw cycles. Food Chem. X.

[55] Zhang J., Chen Q., Liu L., Zhang Y., He N., Wang Q. (2021). High-moisture extrusion process of transglutaminase-modified peanut protein: Effect of transglutaminase on the mechanics of the process forming a fibrous structure. Food Hydrocoll..

[56] Wang Y., Lei A., Zhan Z., Sun X., Zhang F. (2025). Effect of transglutaminase treatment on the physicochemical properties and structural characteristics of soy protein isolate/konjac glucomannan complex. Food Chem..

[57] Renzetti S., Dal Bello F., Arendt E.K. (2008). Microstructure, fundamental rheology and baking characteristics of batters and breads from different gluten-free flours treated with a microbial transglutaminase. J. Cereal Sci..

[58] Zhou P., Labuza T.P. (2007). Effect of water content on glass transition and protein aggregation of whey protein powders during short-term storage. Food Biophys..

[59] Wu C., Wang J., Yan X., Ma W., Wu D., Du M. (2020). Effect of partial replacement of water-soluble cod proteins by soy proteins on the heat-induced aggregation and gelation properties of mixed protein systems. Food Hydrocoll..

[60] Pinho S.C., Brito-Oliveira T.C., Geremias-Andrade I.M., Moraes I.C.F., Gómez-Mascaraque L.G., Brodkorb A. (2024). Microstructure and in vitro digestion of mixed protein gels of soy and whey protein isolates. Food Hydrocoll..

[61] Huang G., Wang Q., Zhong Q., Chen Y., Yang X., Jin W., Xiao G. (2024). Improving color and digestion resistibility of 3D-printed ready-to-eat starch gels using anthocyanins. LWT.

[62] Qiu L., Zhang M., Adhikari B., Lin J., Luo Z. (2024). Preparation and characterization of 3D printed texture-modified food for the elderly using mung bean protein, rose powder, and flaxseed gum. J. Food Eng..

[63] Chen Y., Cai X.-L., Liu L., Zhang T., Qin L.-K., Jia Y.-L. (2025). Preparation and performance characterization of insoluble dietary fiber-alginate-pea protein ternary composite gels. Food Hydrocoll..

