# Supplementary material for: Texture-Modified Soy Protein Gels Using Transglutaminase and Agar for Elderly Dysphagia Management
Source: Gels. 2025 Apr 20;11(4):303. doi: 10.3390/gels11040303 (PMC12026642; doi:10.3390/gels11040303)
Supplement: Supplementary file 1 [file gels-11-00303-s001.zip › gels-3590082-supplementary.pdf]

Supplementary

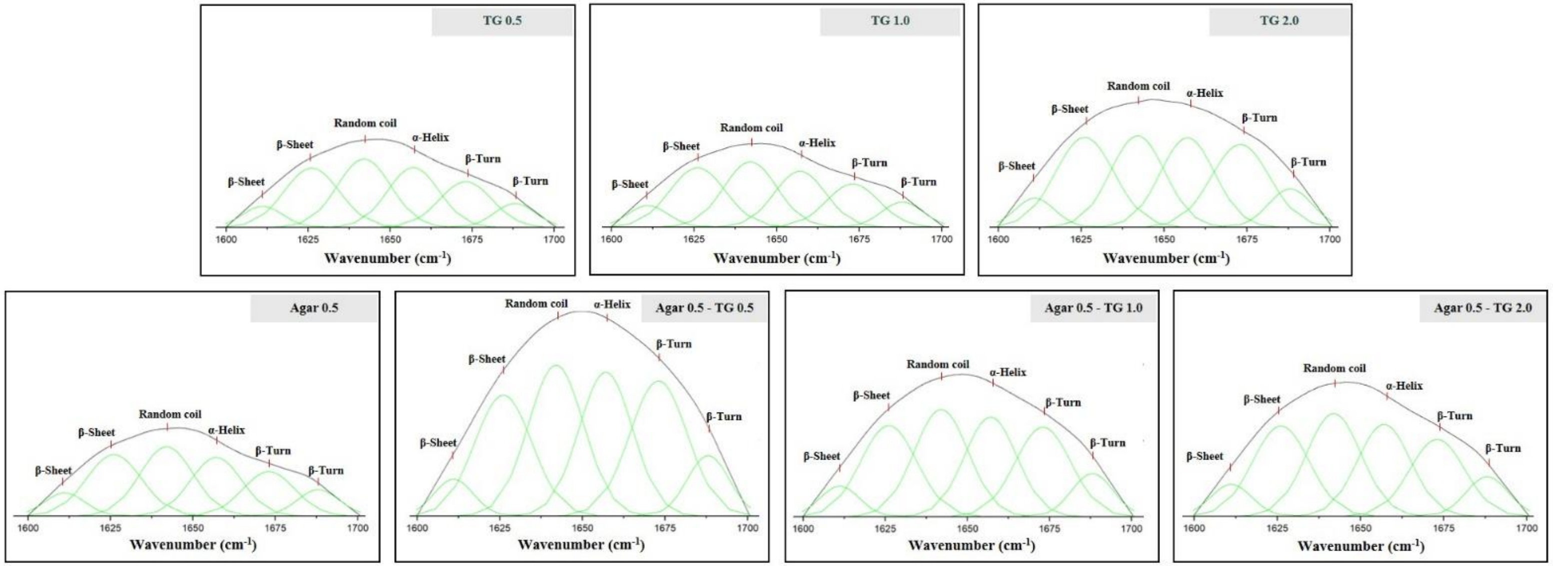

Figure S1. FTIR and deconvolution spectra in the amide I region of soy protein gels
